# New York Medico-Chirurgical College—Morbus Coxarius; Rudimentary Tooth Involuted through the Meatus Auditorius Externus; Cystic Degeneration of the Kidneys; Action of Anæsthetics on Horses; Case of Extra-Uterine Gestation

**Published:** 1860-05

**Authors:** 


### PROCEEDINGS OF SOCIETIES.

*New York Medico-Chirurgical College.* Regular Meeting, February 8, 1860 Dr. Joseph Wooster, Chairman.

Dr. Carnochan presented for examination a patient who had been under his treatment for some time, for *morbus coxarius,* which he said would illustrate in some degree the remarks he had made on this subject at the last meeting.

While the patient was being examined, Dr. Carnochan gave the history of the case, as follows:

This child has passed through all the stages of the disease, and yet the joint is in a pretty good condition, if not entirely well. About three years ago an issue was inserted in the vicinity of the joint, and a mild, general course of treatment adopted. By this treatment, the child was benefited considerably; but after a while, some six or eight months, it relapsed to its former condition, and the disease went on progressing, until finally, the second stage of the disease was reached. At this time, there was an abscess in the neighborhood of the joint, which was punctured with the trochar, and about two tea-cups full of matter allowed to escape.

The child was then placed on the syrup of the iodide of iron, together with the application of the perchloride of iron upon the outside of the joint. After this, the child was taken into the country, it being able to move about on crutches, and it was not long after this before the flow of matter entirely subsided, and the child has now recovered to such an extent, that it is able to walk quite well on the affected side.

It was just such a case as this that I had in my mind when I objected to the universal exsection of the head of the femur in the latter stages of this disease, still not denying that the operation performed at the proper time might be followed by good results.

In fact, I have seen cases in the second stage, where, by using such mild therapeutic measures as the internal use of the iodide of iron, and the external application of the perchloride of iron, at the same time allowing the patients to take exercise on their crutches, they get well with a very good motion of the joint.

Dr. Sayre remarked, that in this case there was no motion whatever in the hip-joint; but what little motion the child had, was confined entirely to the pelvis. We often deceive ourselves in regard to this point, and attribute the motion to the hip-joint. If the members will watch that child closely as it walks across the room, I think they will admit that the motion in this case is confined entirely to the pelvis. It will also be seen that the flexor muscles of the thigh, the tensor vaginæ femoris, the pectineus, the sartorius, are still strongly flexed, although the disease is nearly terminated. I think, then, the case would be very much benefited by the division of the flexor muscles, as it would give the patient the chance, at least, of the formation of a false joint, giving more motion than we now have.

The remarkable power of nature to form false joints, giving excellent motion to the limb, is beautifully illustrated in this specimen, the head of the humerus—which was exhibited to the College. The specimen was removed from a gentleman who received a fracture at this part of the bone some time ago. There was much difficulty in making out the diagnosis, the gentlemen in attendance not believing that any fracture whatever was present; the case, therefore, went on until finally the man died, and the opinion I had expressed at the time of the accident, namely, fracture, was confirmed by a post-mortem examination. We find in this specimen, the fractured end of the bone rounded off and tipped with cartilage, and the cavity in which it rested even lined with *synovial membrane.*

Dr. Carnochan remarked, that he did not suppose from this case, that the same thing would take place in the hip-joint in morbus coxarius. In the case referred to by Dr. Sayre, the joint was in a *healthy* condition; nature had a sound, healthy surface to work on. But in morbus coxarius, we have a morbid condition; the chances for the formation of a false joint are by no means the same in both cases. Still, if we dare run the risk of the child dying from the operation, and are willing to take the responsibility, it is all very well; I am inclined to think, however, that it is far better "to let well enough alone." If we know a case will get along well, we must not run the risk of an operation.

Now, with regard to incision of the muscles, I suppose that Dr. Sayre, of course, divides the muscles subcutaneously; and this is no trivial or easy operation. We have large nerves and vessels in their immediate vicinity, which we must be extremely careful not to wound. Where the muscle is in relief, and is hard and corded, it may, perhaps, be done; but in this case, where the muscles are but little flexed, it would be a somewhat difficult matter to direct the tenotome so as to divide nothing but the muscle.

Dr. Sayre then presented a specimen having the appearance of some two or three molar teeth, half formed, and fused together. They were discharged from the ear of a little girl who visited his office that afternoon. The history of the case was so curious and interesting, that he had requested her to remain, that the members might have an opportunity of examining her. The history is as follows:

Elizabeth ——, aged 8 years, had the measles in Scotland when she was 5 years of age, which was followed by swelling in the neck and all of the right side of the face. The mouth was nearly closed by the swelling of the gums and roof of the upper jaw. The ear was raised upward, and pushed outward by an immense swelling in the region of the mastoid process of the temporal bone. An abscess formed, and was finally opened just below the ear. A large amount of pus escaped, and after about three months she had another febrile attack, followed by intense pain in the ear, which was relieved by a rupture of the tympanum, and the discharge of an immense quantity of pus from the ear. This offensive discharge kept up for about eighteen months, when a *rudimentary tooth* escaped through the *meatus auditorius externus,* which is now in the possession of Dr. Watson, of this city. About four weeks after this, the present specimen was discharged. This specimen is about 1¼ inch long, a half inch wide, and about a half inch thick. It appears to consist of the three posterior molar teeth of the upper jaw. In two weeks after this another portion was discharged, which is about three-quarters of an inch long, half an inch wide, and one quarter of an inch in thickness. This appears to consist of the two posterior molar teeth of the lower jaw. After the escape of these bodies, she was relieved of the pain in a very great measure, and is now perfectly well, but has no hearing on the affected side.

Dr. Nelson remarked, that in examining this child at the present time, it was quite easy to see that all the milk teeth are still remaining; consequently the specimens here exhibited cannot belong to those primitive teeth. Whence, then, come these teeth? There is but one way by which we can account for this strange phenomenon, and that is, by supposing it to be the result of the process of involution. We see so many of these cases where there is a fœtus within a fœtus, or at least a small portion of a fœtus within another, that this manner of accounting for the appearance of these specimens is not rendered improbable. The portion involuted sometimes grows to a certain extent, or the development is arrested quite early. The individual in whom these portions are contained sometimes grows up to an advanced age, and then the portion involuted may be discharged by ulceration, or it may remain inclosed in a cyst and never produce any trouble.

Dr. Meier stated that formations of this kind were not unfrequently found in the interior of ovarian cysts.

Dr. Clark inquired if any one had seen these pieces come out.

The mother of the child, who was present, stated that she was the only one who was by when the pieces came from the ear. In the first instance, her attention was called to the child by its screaming from pain in the ear, directly after getting up. On looking into the ear, she observed a whitish substance, which she supposed was a bit of cotton which had been placed there the evening previous; on taking it out, however, it proved to be a tooth. The expulsion of the second specimen was attended by the same symptoms; so far as she could judge, these teeth seemed to come directly from the external auditory canal.

Dr. Sayre then presented a specimen of extensive *cystic degeneration of the kidneys*, with a written history:

Dr. W. H——, aged 39, of large and robust frame, had slight hæmorrhage from the urethra about eleven years ago, accompanied with intense pain in the region of the right kidney. The blood continued to pass in clots for several days, accompanied with great pain. He was leeched, and bled from the arm very freely, for three days in succession. Was confined to his bed about six weeks, and convalesced slowly in about six months, when he went to California for his health, had the Chagres fever, which prostrated him very much, and left him with a diarrhœa, which continued for nearly eighteen months, the passages, most of the time, being more or less mixed with blood.

For the past two years, he has been compelled to pass his water nearly every half hour, day and night; his wife says he never got up less than eight times in the night for this purpose, and more frequently ten or twelve. He never complained of pain, and only suffered from intense thirst; always drinking a tumbler of water every time he got up to pass his urine, and would drink several pitchers full in the course of the day. The only other symptom that was noticed was a constant and intolerable itching of the anus, which gave him great annoyance, ever since his first hæmorrhage, eleven years since.

Within the past year, he has complained of fatigue and weariness, desiring constantly to rest in a horizontal posture. He, however, continued at his profession—(that of a dentist)—but would frequently leave his patients to rest a few minutes on the sofa; and which he attributed to his growing so fat, and was surprised to find that his increase in size was confined entirely to his waist.

Whenever he used the furnace to bake his porcelain, for the past year, he has suffered from epistaxis, which continued for several days, generally a week. Within the last year he has had a great many boils on different parts of the body; his wife thinks more than a hundred, from which he suffered great pain.

On Monday, the 9th of January, (1860,) he complained of a pain in the region of the right kidney, for the first time in ten years. This continued to increase until the 14th, when it became very severe, and about 3 o'clock he laid down, and opened his pants, complaining of their tightness, and, pressing both hands on his loins, asked his wife to rub and *press* him there, when he suddenly jumped up, crying out, " There, something has given way—now it's coming," and immediately the blood started, and he passed nearly three quarts. I saw him a few hours afterwards in consultation with Dr. Senff, and found him bathed in a cold, clammy sweat; pulse 130, small, easily compressed, and feeble; complaining of pain and weight in the right kidney. He was under the influence of morphine, and did not evacuate his bladder for thirty-two hours, at which time he passed about one pint of blood, dissolved in a small quantity of urine. Whether the check of secretion from the kidneys was due to the excessive loss of blood, or the use of morphine, I was unable to determine. He continued to pass bloody urine for three or four days, when it again became clear and transparent, of the same amount, unmixed with blood. On the 20th, he had a slight convulsion, which was followed by a copious hæmorrhage and increased pain in the right kidney. A large-sized catheter passed readily into the bladder, showing there was no obstruction in the urethra; but the bladder was very small and corrugated, giving a very roughened feel to the hand when describing a circle with the instrument.

A distinct tumor could be detected in the right side, commencing just above the ilium, and extending upward, in the region of the kidney. It had an elastic, fluctuating feel, like an intestine filled with air, but was not resonant upon percussion, and was therefore diagnosticated, as being connected with the kidney. He died in a convulsion on the 30th January, 1860, and on a *post-mortem*, is found the following condition of the kidneys:

Right, weighed 4 lbs. $\frac{3}{4}$ oz.; length, $13\frac{1}{2}$ in.; breadth, 7 in.; thickness, 4 in. Left, weighed 3 lb. $\frac{1}{2}$; length, 11 in.; breadth, $5\frac{1}{4}$ in.; thickness, $3\frac{1}{4}$ in. Made up of cysts, varying in size from a large walnut to a small pea, and filled with different-looking fluid—from a transparent serum, amber-colored, quince juice, jelly-looking material, up to the purple and jetty black; the latter, however, consisting mostly of blood. All of the black and bloody cysts were on the right side; the left was apparently transparent serous cysts. Both had undergone to a considerable extent the fatty degeneration. The bladder was very small and hypertrophic in its muscular coat; mucous membrane slightly thickened, but no hæmorrhagic spots. All the other organs were properly healthy. The lungs were much compressed by the distention of the kidneys, but were healthy.

The brain was not examined.

Dr. Bryant remarked, that with the permission of the Society, he would read a letter which he had received from Dr. Jennings, Veterinary Surgeon, of Philadelphia, in answer to a request for his experience with regard to the *action of anæsthetics on horses.*

The letter gave the reports of twenty-one cases, in some of which chloroform was given, for the purpose of destroying life; but in most, to facilitate the removal of tumors and the performance of other operations.

The reporter stated that he had never known a case terminate fatally, from the use of chloroform in the horse, unless it was so designed. Sulphuric ether he regarded as of no service whatever, as an anæsthetic in operations on the horse; chloric ether answers very well, but cannot be depended upon. He had had no experience as to its effects on dogs, having used it in one instance only, that of a water spaniel; but the dog died within three minutes after the first administration of the anæsthetic. He had been informed by a friend, who had experimented much upon dogs, that with these animals it was a dangerous agent, terminating almost always fatally.

Dr. Carnochan remarked, that the very rapid manner in which the horses died under the influence of chloroform, was somewhat remarkable. There are several cases mentioned, where the animals died in some sixteen or seventeen minutes. In the human subject, he had frequently seen patients kept under the influence of the anæsthetic as long as an hour without any injurious result following.

Dr. Bryant.—The rapid destruction of life in the cases referred to, must be attributed to the fact, that the agent was administered for the purpose of producing death, and therefore, no precaution was taken to admit the atmosphere; on the contrary, the admission of air was almost entirely prevented, so that in addition to the influence of the anæsthetic, the animal was subjected to suffocation.

Dr. Wooster stated, that in one instance he had purposely destroyed the life of a horse in this manner, and it was only with the greatest difficulty that the animal could be brought under the influence of the anæsthetic. Some eight ounces of chloroform were used; the animal lying in a recumbent position. On raising up the head for a few minutes, death soon took place. He thought that the injurious results seen in the human subject might often be attributed to suddenly raising the patient to a semi-erect posture, thus depriving the brain of its arterial stimulus.

Dr. Bronson presented a *fœtus*, which was the product of *extra-uterine gestation*, being delivered through a spontaneous opening in the abdominal walls, with the following history: On Wednesday, the 25th of January, I was summoned to see a patient, from whom I learned that she had conceived in April, 1859; that she had had peculiar sensations while carrying the child, but especially during the first part of December, about the 12th of which month, the child ceased to move, and since that time, she has been gradually failing. The peculiar sensations she spoke of were those of *crawling* from right to left; a sensation which is easily accounted for, as will be seen hereafter. Having heard this much of the history, I made a partial examination of her case. I found the abdomen enlarged naturally, as from pregnancy, but to a limited extent; and in the course of the colon, the abdomen was enlarged, painful, and tympanitic. On pressing upon the parietes gently, but firmly, a mass was felt, simulating fæcal accumulation, and such I supposed it to be, and in part, rightly so. The patient was then in a hectic condition, pale and emaciated. Pulse 156. The surface of the skin, as well as the respiratory mucous membrane, gave forth a very peculiar odor. The cervix uteri did not present that condition which is ordinarily found in pregnancy at this stage, which I attributed to the fact that the fœtus had been long dead. Such being the condition of the patient, I resolved upon the following course of treatment : To give quinine and morphine at intervals of three hours, together with brandy *ad libitum*, and the most nourishing diet. In addition to this, an enema was ordered night and morning, with the intention, as soon as her strength would permit, of exploring the uterus, and removing the source of her trouble. In the course of four days, her strength improved and the pulse became reduced to 128, but of little power. On the fourth and fifth days, I was enabled, by means of sponge tents, to examine the uterine interior, which I found empty. During the first four days, the injections brought away large quantities of compact fæcal matter, after which, the tumefaction in the course of the colon was materially reduced. At a point, however, midway between the umbilicus and the borders of the ribs upon her left side, the pain and tenderness increased, and the skin in that region became heightened in color. Her strength did not maintain its apparent increase, but gradually failed. On the 6th of February, at a point three inches and a half above the umbilicus, and one inch and a half to the left of the median line, the parietes of the abdomen had become so far absorbed as to present a small aperture, through which fœtid and intestinal gases escaped. On the 9th of February, which is to-day, the aperture was found to be enlarged, simply by this process of absorption, to the diameter of one and a half inch, and through this opening, in the presence of, and assisted by, Dr. H. G. Davis and my pupil, Mr. Anderson, I removed a full-grown fœtus, the soft parts of which are, to a great extent, decomposed; the fibrous and osseous tissue are still very perfect, presenting, as is seen in the specimen, the form and appearance of a fœtus. The fœtus was lying with its feet in the right iliac fossa, and the head in the left, the postero-lateral portion of the thorax of the left side presenting at the opening. This position accounts for the crawling motion felt by the mother during the life of the fœtus. No placenta could be found, it having probably passed away through an opening which, I should have stated before, was found in the colon, directly opposite the external opening. After the removal of the fœtus, the mother was put upon quinine and morphine, brandy, &c., and left to rest.

Dr. Bryan remarked, that he knew of two cases that were analogous to this in some respects. One of them occurred in the practice of Dr. Yardly, of Philadelphia. In this instance, the fœtus was removed, piece-meal, from the anus of the patient. It was supposed that it made its way from the posterior wall of the uterus, by ulceration into the rectum; the exact nature of the case, however, was not clearly ascertained.

The other case occurred in the practice of Dr. Bryan's brother, at Beverly, N. J. The patient was a fine, healthy woman, in every respect; she was taken in labor with the usual pains, when, very suddenly, the labor ceased; she recovered from the immediate effects of this shock. A tumor, however, still remained in the abdomen, and continued in this situation for some six months, until finally ulceration took place, and the fœtus was discharged per rectum.

